# Proton Pump Inhibitor Use and Its Association with Lung Cancer Likelihood and Mortality: A Nationwide Nested Case–Control Study in Korea

**DOI:** 10.3390/cancers17050877

**Published:** 2025-03-04

**Authors:** Mi Jung Kwon, Ho Suk Kang, Hyo Geun Choi, Joo-Hee Kim, Ji Hee Kim, Woo Jin Bang, Dae Myoung Yoo, Na-Eun Lee, Kyeong Min Han, Nan Young Kim, Sangkyoon Hong, Hong Kyu Lee

**Affiliations:** 1 Department of Pathology, Hallym University Sacred Heart Hospital, Hallym University College of Medicine, Anyang 14068, Republic of Korea; mulank99@hallym.or.kr; 2 Division of Gastroenterology, Department of Internal Medicine, Hallym University Sacred Heart Hospital, Hallym University College of Medicine, Anyang 14068, Republic of Korea; hskang76@hallym.or.kr; 3 Suseo Seoul E.N.T. Clinic, 10, Bamgogae-ro 1-gil, Gangnam-gu, Seoul 06349, Republic of Korea; mdanalytics@naver.com; 4 Division of Pulmonary, Allergy and Critical Care Medicine, Department of Medicine, Hallym University Sacred Heart Hospital, Hallym University College of Medicine, Anyang 14068, Republic of Korea; luxjhee@hallym.or.kr; 5 Department of Neurosurgery, Hallym University Sacred Heart Hospital, Hallym University College of Medicine, Anyang 14068, Republic of Korea; kimjihee@hallym.or.kr; 6 Department of Urology, Hallym University Sacred Heart Hospital, Hallym University College of Medicine, Anyang 14068, Republic of Korea; yybbang@hallym.or.kr; 7 Hallym Data Science Laboratory, Hallym University College of Medicine, Anyang 14068, Republic of Korea; ydm@hallym.ac.kr (D.M.Y.); intriguingly@hallym.ac.kr (N.-E.L.); km.han@hallym.ac.kr (K.M.H.); 8 Laboratory of Brain and Cognitive Sciences for Convergence Medicine, Hallym University College of Medicine, Anyang 14068, Republic of Korea; 9 Hallym Institute of Translational Genomics and Bioinformatics, Hallym University Medical Center, Anyang 14068, Republic of Korea; honeyny@hallym.or.kr (N.Y.K.); kyoons@hallym.or.kr (S.H.); 10 Department of Thoracic and Cardiovascular Surgery, Hallym University Sacred Heart Hospital, Hallym University College of Medicine, Anyang 14068, Republic of Korea

**Keywords:** lung cancer, proton pump inhibitors, risk assessment, survival, nested case–control study, national healthcare data, big data analysis

## Abstract

Background/Objectives: Proton pump inhibitors (PPIs) are widely used for acid-related gastrointestinal disorders, but their potential association with lung cancer risk and mortality remains underexplored and debated. This study sought to investigate the association between PPI use and lung cancer likelihood and mortality, focusing on the impact of PPI exposure history and duration. Methods: This study utilized data from 6795 lung cancer patients, 27,180 matched controls, and 4257 deceased and 2538 surviving lung cancer patients from the Korean National Health Insurance Service’s Health Screening Cohort (2002–2019). Propensity score overlap weighting and logistic regression models were applied to assess the correlations between PPI usage history and duration with lung cancer risk and mortality, while standardized differences ensured balanced baseline characteristics. Results: Overall, PPI use was modestly associated, with a 19% increased likelihood of lung cancer occurrence (95% confidence intervals (CI): 1.12–1.26). Interestingly, prolonged PPI use (≥30 days) was linked to a 13% reduction in lung cancer incidence (95% CI: 0.80–0.94), particularly in subgroups such as older adults (≥70 years), individuals with gastroesophageal reflux disease (GERD) or hypertension, and those with low alcohol consumption. Conversely, overall PPI usage was linked with a 36% increased mortality likelihood among lung cancer patients (95% CI: 1.20–1.55), with prolonged use further correlating with a 27% higher mortality risk (95% CI: 1.05–1.53), especially in high-risk subgroups, including smokers, underweight individuals, and those with hypercholesterolemia or GERD. Conclusions: These findings may suggest a complex and context-dependent relationship between PPI use and lung cancer outcomes, emphasizing the need for individualized risk assessments and careful prescribing practices.

## Simple Summary

This research investigates the potential link between proton pump inhibitors (PPIs) and lung cancer risk and survival in the Korean population. Using a large dataset from the Korean National Health Insurance Service, we analyzed over 6700 lung cancer patients and compared them with over 27,000 matched controls. The results indicate that prolonged PPI use may reduce lung cancer risk in specific groups, such as older adults and individuals with certain health conditions, but it may increase mortality in vulnerable populations like smokers or underweight patients. These results emphasize the need for personalized approaches to prescribing PPIs. This research contributes to understanding the nuanced effects of PPIs on lung cancer outcomes and may guide safer clinical practices in their use.

## 1. Introduction

Proton pump inhibitors (PPIs) are widely prescribed for managing acid-related gastrointestinal disorders, such as gastroesophageal reflux disease (GERD), peptic ulcers, and functional dyspepsia, due to their efficacy in suppressing gastric acid secretion [1]. These medications have significantly reduced hospitalizations and mortality associated with upper gastrointestinal complications, contributing to their widespread adoption in clinical practice globally [1], including a 1.5-fold increase in their usage in Korea over the past five years [2].

Despite their proven benefits, concerns regarding the potential adverse effects of PPIs have grown, particularly with long-term use [3,4]. These include risks such as nutrient malabsorption, osteoporosis, kidney damage, enteric infections, pneumonia, and increased susceptibility to certain cancers [5,6,7,8], including gastric [9], esophageal [10], colorectal [11], and pancreatic cancers [12]. However, the relationship between PPI use and lung cancer remains insufficiently studied, with limited and conflicting evidence [13,14]. While one Korean study suggested that certain medications, including PPIs, may reduce lung cancer risk, it had methodological limitations, notably combining PPIs with H2 receptor antagonists, complicating the distinction of each drug’s specific effects [13]. Conversely, a U.S. study indicated an increased lung cancer risk in nonsmokers with GERD [14], although it did not directly evaluate the effects of PPIs.

Several hypotheses suggest potential mechanisms through which PPIs may contribute to lung cancer development. These include their impact on systemic inflammation, alterations in the gut–lung axis, and modulation of immune responses. PPIs can disrupt the gut and respiratory microbiome, which has been linked to carcinogenesis [15]. Prolonged use may also lead to chronic esophageal and airway inflammation caused by acid reflux—a recognized precursor to lung cancer development. Additionally, PPIs can interfere with the metabolism of certain anticancer drugs by altering the gastric pH, which may reduce drug efficacy [16]. Genetic polymorphisms affecting PPI metabolism, particularly among East Asians with reduced CYP2C19 activity, may contribute to prolonged drug exposure and influence the cancer risk [17,18].

PPIs are frequently prescribed to cancer patients to manage symptoms such as reflux, often forming part of complex treatment regimens [19]. However, their potential impact on cancer outcomes appears to vary across cancer types. For instance, PPI use has been shown to have beneficial effects in melanoma but negative effects on survival outcomes in non-small cell lung cancer [16]. These discrepancies may be partially attributed to differences in genetic polymorphisms affecting PPI metabolism [17,18], particularly among East Asians, who are more likely to be poor metabolizers of PPIs due to reduced CYP2C19 activity [18,20]. This slower metabolism could lead to prolonged drug exposure and potentially influence both the therapeutic and adverse outcomes.

Given the rising prevalence of GERD and lung cancer in Asia [21,22] and the widespread use of PPIs [23], understanding the nuanced relationship between PPI use and lung cancer outcomes is critical. This study aims to evaluate the likelihood of lung cancer occurrence and mortality among lung cancer patients based on their prior PPI exposure history and duration, leveraging a nationwide cohort from the Korean National Health Insurance Service. By employing advanced statistical methodologies and subgroup analyses, we provide insights into the context-dependent effects of PPI use and their implications for personalized treatment strategies.

## 2. Materials and Methods

### 2.1. Data Source

This study utilized data from the Health Screening Cohort of the Korean National Health Insurance Service (KNHIS-HSC), collected between 2002 and 2019, which include sociodemographic characteristics, healthcare utilization, health screening results, and dates of birth and death for the entire population of South Korea [24]. The database is de-identified by the government and uses the International Classification of Diseases, 10th revision (ICD-10), codes for healthcare information and standardized disease diagnosis.

The present study was approved by the ethics committee of Hallym University (2019-10-023). This study used the KNHIS-HSC data from 2002 through 2019, which were collected in the Korean National Health Insurance Service; thus, written informed could not be achieved and was exempted by the ethics committee.

### 2.2. Exposure (Proton Pump Inhibitor)

We retrospectively analyzed the PPI prescription records from one year preceding the diagnosis of lung cancer within the cohort groups. Participants were eligible for inclusion if their initial PPI use occurred within a year prior to the index date. The index date refers to the date when the corresponding ICD-10 code C34 was first recorded in the health insurance claims database, indicating the initial diagnosis of lung cancer. PPI usage history was categorized into three groups: non-users, current users (prescribed at least once within the last 30 days), and past users (prescribed at least once between 31 and 365 days prior). For analysis, current and past users were combined into a single “user” category to assess the temporal relationship between PPI exposure and the likelihood of cancer. The cumulative duration of PPI use was assessed by summing the total prescription days within the year before the index date and categorized into two groups: <30 days and ≥30 days.

To minimize the potential for selection bias in defining PPI exposure, we applied several strategies. First, we categorized the PPI exposure duration into distinct groups (<30 days and ≥30 days) to differentiate short-term users from those with prolonged exposure, which more likely reflects chronic use rather than transient prescriptions. Second, we employed propensity score overlap weighting to balance the baseline characteristics between groups, reducing the influence of confounding factors such as underlying health conditions and comorbidities that may lead to PPI prescriptions.

Additionally, to address potential reverse causality, we excluded individuals with a diagnosis of lung cancer within the first year of the study period. This exclusion helped minimize the likelihood that early, nonspecific symptoms of lung cancer could have prompted transient PPI use. Our subgroup analyses further explored the associations across different levels of comorbidity burden and PPI use duration, providing a deeper understanding of these complex relationships.

### 2.3. Outcome (Lung Cancer)

Lung cancer was designated based on the ICD-10 code C34 having been assigned to more than three clinic visits from 2002 through 2019. For the accuracy of diagnosis, we only chose people who had visited the clinics ≥3 times since the diagnosis of lung cancer. Among them, we selected lung cancer participants who underwent surgery or chemotherapy or radiotherapy.

The primary outcome assessed was the likelihood of lung cancer occurrence based on PPI use history (never vs. exposed) and usage duration (<30 days vs. ≥30 days). The secondary outcome examined the odds of overall or all-cause mortality among these patients in relation to PPI use.

### 2.4. Cohort Selection

**Figure 1:**
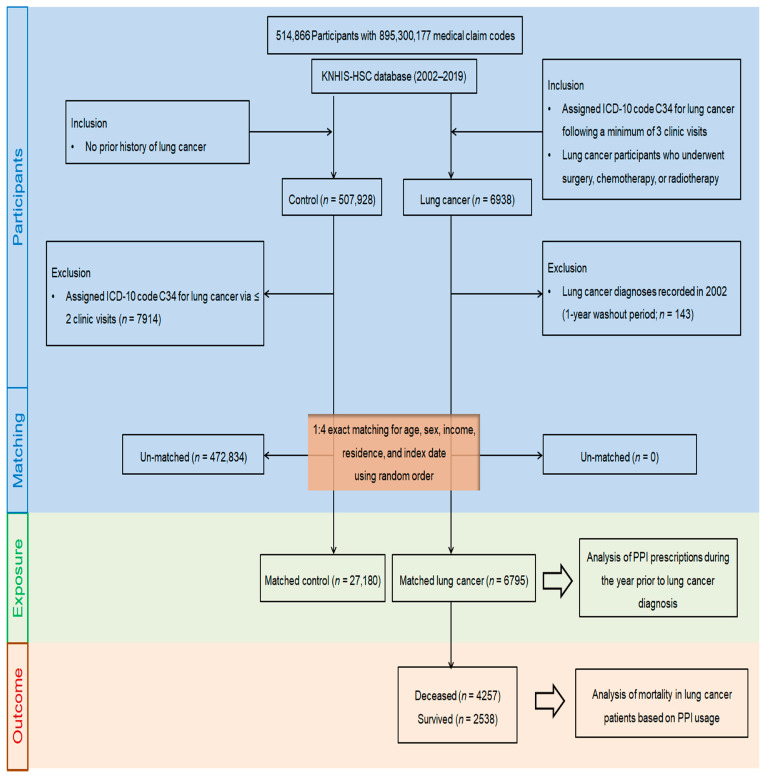
A schematic representation of the participant selection process in this study. From the initial cohort of 514,866 individuals, 6795 lung cancer patients and 27,180 matched controls, as well as 4257 deceased and 2538 surviving lung cancer patients, were identified and included using data from the Korean National Health Insurance Service’s Health Screening Cohort (KNHIS-HSC) (2002–2019).

A total of 6938 newly diagnosed lung cancer patients from 2002 to 2019 were identified from the KNHIS-HSC database, which includes 514,866 participants aged over 40 years and 895,300,177 medical claim records (Figure 1). To minimize false-positive cases, lung cancer diagnoses were identified using ICD-10 code C34, requiring at least three clinic visits for diagnostic confirmation. Patients who were diagnosed with lung cancer in 2002 (n = 143) were excluded to ensure a 1-year washout period, with the index date being defined as the first recorded ICD-10 code C34 in the health insurance database.

The control cohort consisted of individuals who had never been diagnosed with lung cancer between 2002 and 2019 (n = 507,928). Control participants were excluded if they had been diagnosed with lung cancer (C34) but had fewer than three clinic visits for diagnosis (n = 7914).

To ensure balanced baseline characteristics between lung cancer patients and control participants, propensity score matching was performed based on age, sex, income, and residence, employing random clustered sampling to mitigate potential selection bias. The index dates for control participants were matched to the index dates of their corresponding lung cancer patients, ensuring identical reference points for comparison. Through the matching steps, 472,834 control members were eventually unmatched and eliminated, whereas no lung cancer cases were unmatched during this process. Therefore, 6795 patients with lung cancer were matched with 27,180 control members at a ratio of 1:4. Lung cancer participants were stratified into deceased (n = 4257) and surviving patients (n = 2538) to evaluate mortality outcomes following a lung cancer diagnosis in relation to PPI use history and duration.

### 2.5. Covariables

Participants were categorized by age (10 groups) and income level (5 tiers), with residential areas being classified as urban or rural. Lifestyle factors, including smoking, alcohol consumption, and body mass index (BMI), were analyzed alongside physiological measures such as blood pressure, fasting glycemic values, and total cholesterol. The Charlson Comorbidity Index (CCI) was used to quantify the overall disease burden, assigning scores (0–29) based on the presence and severity of 17 comorbidities, offering a standardized assessment of cumulative health impacts [25].

### 2.6. Statistical Analyses

To address potential confounding factors and achieve a balanced comparison between lung cancer patients and controls, we utilized propensity score overlap weighting. This method assigns weights to participants based on the probability of their group membership (lung cancer or control) given the baseline characteristics [26,27]. Unlike traditional propensity score matching, overlap weighting emphasizes the region of common support between groups, thereby minimizing bias and maximizing the effective sample size [26,27].

Specifically, propensity scores were calculated using a logistic regression model incorporating baseline covariates, including region, income, age, BMI, blood glucose, cholesterol, blood pressure, and comorbidities (CCI score) [28]. Overlap weights were then derived, assigning higher weights to participants with similar probabilities of being in either group, thus ensuring better comparability [26,27]. This method allowed us to create a well-balanced dataset, as evidenced by the standardized differences in covariates before and after weighting. A standardized difference of less than 0.20 was considered indicative of an acceptable balance [29]. The overlap-weighted dataset was subsequently analyzed using logistic regression models to estimate odds ratios (ORs) and 95% confidence intervals (CIs) for lung cancer risk and mortality based on PPI usage history and duration. Subgroup analyses were conducted across all variables. Statistical analyses were performed using SAS 9.4, with significance defined at *p* < 0.05.

## 3. Results

### 3.1. Baseline Characteristics of Participants

The baseline features of participants before and after propensity score overlap weighting are summarized in Supplementary Table S1. Before weighting, significant imbalances were observed in several covariates between lung cancer and control groups, including age, smoking status, and body weight. Lung cancer participants were more frequently identified as current smokers and underweight or normal weight compared to controls. After the overlap propensity score weighting, standardized differences were reduced to ≤0.20 for all covariates, achieving an adequate balance between groups.

### 3.2. PPI Use History and Incident Lung Cancer Risk

The relation between PPI use history and the risk of incident lung cancer is summarized in Table 1. Crude analyses demonstrated a significant connection between PPI use history and lung cancer (OR = 1.79; 95% CI: 1.66–1.91; *p* < 0.001). After adjustment with overlap weighting, the association weakened but retained statistical significance (adjusted OR = 1.19; 95% CI: 1.12–1.26; *p* < 0.001).

The subgroup analyses (Supplementary Table S2) confirmed that the association between PPI intake history and lung cancer likelihood was robust and consistent across various subgroups, including different age groups, income brackets, residential regions, smoking and alcohol drinking statuses, pressure levels, fasting glycemic values, or total cholesterol levels. Moreover, associations were observed among male participants, non-obese individuals, and those with a CCI score ≥ 2. Among non-GERD participants, PPI use history was strongly associated with subsequent occurrence of lung cancer (adjusted OR = 1.51; 95% CI: 1.39–1.64; *p* < 0.001). Conversely, GERD participants showed a reduced adjusted OR for incident lung cancer (0.65; 95% CI: 0.59–0.72; *p* < 0.001).

### 3.3. Duration of PPI Use and Lung Cancer Risk

The correlation between the duration of PPI prescription and lung cancer likelihood is summarized in Table 1. Crude analyses indicated that prolonged PPI use (≥30 days) was linked with an elevated probability of lung cancer (OR = 1.43; 95% CI: 1.29–1.58; *p* < 0.001). However, after adjustment using overlap weighting, extended PPI use was linked with a significantly lessened probability of lung cancer (adjusted OR = 0.87; 95% CI: 0.80–0.94; *p* < 0.001).

**Figure 2:**
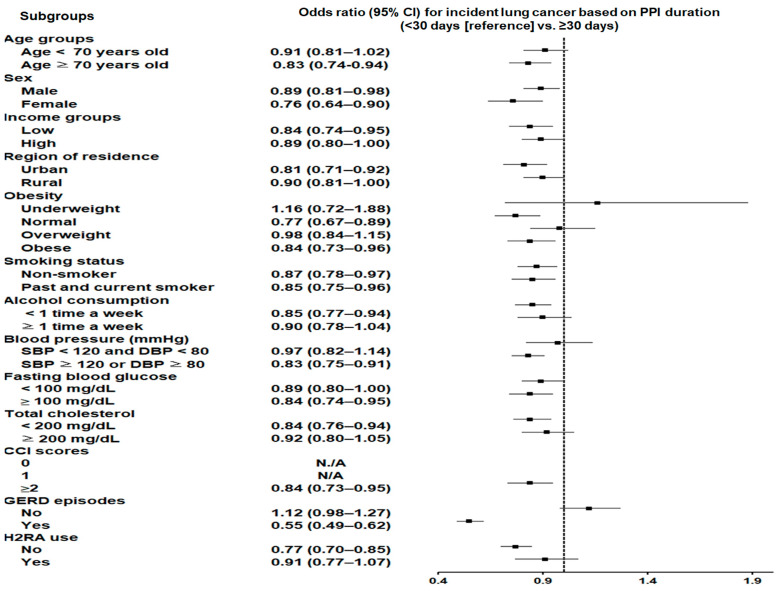
The reduced likelihood of lung cancer with prolonged PPI use (≥30 days) was consistent across sex, income level, residential area, smoking history, and fasting glucose. It was also observed in participants aged ≥70 years, those consuming alcohol less than once per week, individuals with hypertension, and those with total cholesterol levels below 200 mg/dL. Notably, GERD patients showed a consistent reduction in lung cancer risk with prolonged PPI use. Abbreviations: CI, confidence interval; PPI, proton pump inhibitor; SBP, systolic blood pressure; DBP, diastolic blood pressure; CCI, Charlson Comorbidity Index; GERD, gastroesophageal reflux disease; H2RA, H2 receptor antagonist.

In the subgroup analyses (Supplementary Table S3), the reduced likelihood of incident lung cancer associated with longer PPI use was consistent regardless of sex, income level, residential area, smoking history, or fasting glycemic values. A reduced likelihood of lung cancer with longer PPI use was also observed among participants aged ≥70 years, participants consuming alcohol less than once per week, individuals with hypertension, and those with total cholesterol levels below 200 mg/dL. Furthermore, PPI users with GERD showed a consistent reduction in lung cancer risk with prolonged use (Figure 2).

### 3.4. Association Between PPI Use History and Mortality in Lung Cancer Participants

The baseline features of lung cancer participants who were stratified based on mortality status before and after the overlap weighting adjustment are described in Table 2. Participants who succumbed to lung cancer were predominantly older (≥70 years old), male, and of lower income compared to survivors. A higher proportion of deceased participants were underweight or current smokers. Notably, the CCI score was significantly higher among deceased participants compared to survivors.

Participants with a history of PPI use exhibited a significantly elevated probability of mortality compared to non-users (adjusted OR 1.36; 95% CI: 1.20–1.55; *p* < 0.001) (Table 3). The subgroup analyses (Supplementary Table S4) showed that the increased mortality likelihood in relation to PPI use history persisted across diverse subgroups, regardless of sex, economic strata, residential area, alcohol intake, blood pressure, fasting glycemic values, total cholesterol level, or GERD history. Notable findings included a heightened mortality risk among participants aged ≥70 years, those without obesity, past or current smokers, and individuals concurrently using H2 receptor antagonists.

### 3.5. Association Between Duration of PPI Use and Mortality in Lung Cancer Participants

**Figure 3:**
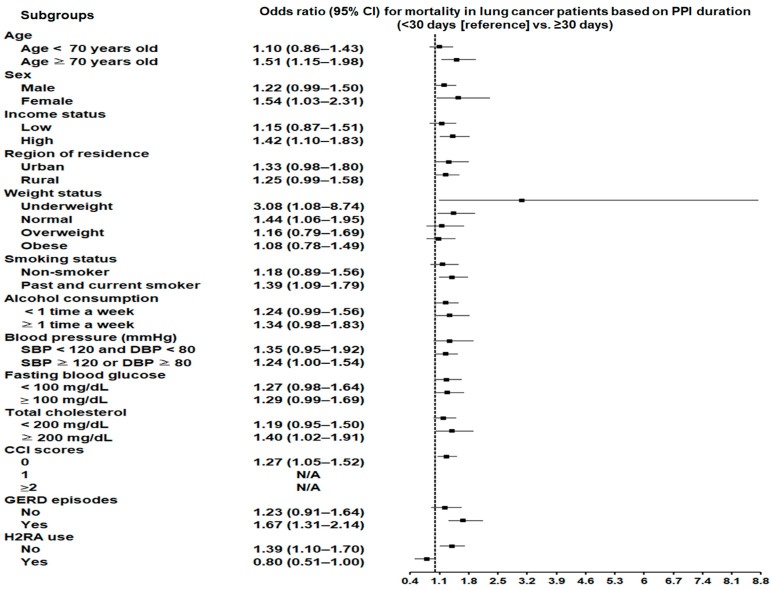
Prolonged PPI use correlated with higher mortality across sex and in participants aged ≥70 years, high-income groups, underweight individuals, past or current smokers, and those with total cholesterol ≥200 mg/dL or GERD. Abbreviations: CI, confidence interval; PPI, proton pump inhibitor; SBP, systolic blood pressure; DBP, diastolic blood pressure; CCI, Charlson Comorbidity Index; GERD, gastroesophageal reflux disease; H2RA, H2 receptor antagonist.

Prolonged PPI use was related to a significantly higher mortality risk compared to shorter durations, with an adjusted OR of 1.27 (95% CI: 1.05–1.53; *p* = 0.012). The subgroup analyses (Supplementary Table S5) showed that prolonged PPI prescription correlated with higher mortality regardless of sex and among participants aged ≥70 years, those in high-income groups, underweight individuals, past or current smokers, and those with total cholesterol levels ≥200 mg/dL or GERD (Figure 3).

## 4. Discussion

This nationwide cohort study revealed a multifaceted relationship between PPI use and lung cancer outcomes, possibly suggesting both its context-dependent risks and potential protective effects. Overall, a history of PPI exposure correlated with elevated odds of developing lung cancer and higher mortality rates among lung cancer patients. However, the duration of PPI use appeared to modify these risks, with prolonged use showing differential effects: a potential protective association with lung cancer development in specific subgroups, such as older adults, individuals with GERD or hypertension, and those with low alcohol consumption was found, while elevated mortality risks were observed in underweight individuals, smokers, and those with hypercholesterolemia or advanced age. These findings may underscore the need for personalized PPI prescription strategies, considering patient demographics, underlying comorbidities, and duration of use. Stratified risk assessments may be crucial to balance the risks and benefits, particularly in populations with varying susceptibilities.

Our study found that overall PPI exposure was modestly associated with a 19% increase in the likelihood of lung cancer occurrence (95% CI: 1.12–1.26) after adjusting for confounders. In the crude analyses, the association appeared stronger (1.79-fold increase; 95% CI: 1.66–1.91); however, this was attenuated following propensity score overlap weighting, which accounted for potential confounding variables. These findings may underscore the critical importance of addressing confounding factors when examining the correlation between PPI use and lung cancer risk. Indeed, prior research has emphasized that factors such as age, sex, lifestyle, obesity, genetic predispositions, and comorbidities may confound the observed associations between PPI exposure and cancer development [30]. By utilizing propensity score overlap weighting, our study employed a robust methodological approach, yielding more reliable estimates of the correlation between PPI administration and lung cancer outcomes.

The subgroup analyses revealed notable differences in the association between PPI use and lung cancer risk across various populations. In patients with GERD, prolonged PPI use (≥30 days) was associated with a significantly reduced likelihood of lung cancer (adjusted OR = 0.55; 95% CI: 0.49–0.62). This protective effect may be attributed to the ability of PPIs to mitigate chronic esophageal and airway inflammation caused by acid reflux [31], a recognized precursor to carcinogenesis [32]. By reducing gastric acid production and associated esophageal damage, PPIs may interrupt inflammation-driven cancer pathways, particularly in GERD patients [12,30]. Our findings partly align with those of a former Korean epidemiological study, which reported that longer antacid use, including PPIs, was associated with reduced lung cancer incidence [13]. Longer antacid exposure across all the different duration categories (14–34 days, 35–77 days, ≥78 days) was associated with a reduced incidence of lung cancer compared to short-term use (<14 days) [13]. Despite using the same Korean healthcare data, our research extended the observation period (2002–2019) and exclusively focused on PPIs, offering a more comprehensive analysis. In contrast, the previous study examined both PPIs and H2 receptor antagonists collectively, with a limited analysis of PPIs’ effects due to a small sample size of PPI users (1.2% of cohort participants) and a shorter study period (2002–2010) [13]. Additionally, our study provided a more detailed estimation of lung cancer risk differences across various clinical and demographic subgroups. The current findings are noteworthy, as they were derived from a homogeneous and evenly balanced cohort of lung cancer cases and matched controls, with adjustments being made using overlap weighting. In this context, the present epidemiological investigation may offer an updated perspective on the potential protective effects of antacid use, particularly PPIs, against lung cancer development, building on prior evidence from the Korean population.

Conversely, among non-GERD participants, PPI use history was linked to an elevated risk of lung cancer (adjusted OR = 1.51; 95% CI: 1.39–1.64). This disparity suggests that in the absence of acid-reducing benefits in GERD, other factors—such as changes in gut microbiota composition or systemic inflammation—might contribute to increased cancer susceptibility [3,30,33]. Experimental evidence has shown that PPIs can disrupt the gut–respiratory microbiome axis, potentially influencing carcinogenesis beyond the gastrointestinal tract [33], which could potentially influence respiratory health [16]. These findings underscore the importance of underlying conditions in modulating the relationship between PPI use and cancer risk.

Our analysis of mortality outcomes indicated that prolonged PPI use was associated with a heightened risk of death in certain high-risk subgroups, including smokers (adjusted OR = 1.39; 95% CI: 1.09–1.79) and underweight individuals (adjusted OR = 3.08; 95% CI: 1.08–8.74), suggesting the need for cautious PPI prescription for prolonged durations in this vulnerable population. Smokers, who are already predisposed to lung cancer and poor survival due to smoking-related carcinogenic effects [34], may experience compounded risks when exposed to PPIs [14]. Mechanistically, PPIs could interfere with the absorption of certain anticancer drugs by altering the gastric pH, potentially reducing their efficacy [35,36,37,38]. Additionally, smokers are more susceptible to infections such as pneumonia, which has been independently associated with PPI use [39] and could further worsen the outcomes in this population.

For underweight individuals, the increased mortality risk might be linked to poor baseline nutritional status and systemic vulnerabilities that amplify the adverse effects of PPIs, such as infections or impaired immune responses [40]. Similarly, participants with hypercholesterolemia (adjusted OR = 1.40; 95% CI: 1.02–1.91) exhibited elevated mortality, potentially due to the interplay between lipid metabolism, systemic inflammation, and cancer progression [30,37].

The direct mechanisms underlying the link between PPI usage and lung cancer likelihood or survival remain poorly understood. However, experimental studies provide some clues, highlighting the dual properties of PPIs that may vary depending on the cancer cell type [16,41,42]. In vitro and in vivo studies have demonstrated variable effects of V-ATPase activity, with both tumor-promoting and tumor-suppressing impacts being observed in malignancies such as gastric, esophageal, pancreatic, breast, cervical, and prostate cancers and malignant melanomas [16,42]. One proposed mechanism is that PPIs can modulate the tumor microenvironment by increasing intracellular acidification, reducing acid-mediated inflammation, and directly inhibiting the activity of proton pumps of V-ATPase in certain cancer cells [41], which may contribute to tumor progression under specific conditions. Additionally, since PPIs are metabolized by hepatic cytochrome P450 enzymes [20], individuals with genetic polymorphisms that impair enzyme activity, as seen more frequently in the Korean population, may experience higher systemic levels of PPIs with short-term or low-dose use [20,43], of which genetic polymorphisms may partly explain the increased likelihood of lung cancer observed among overall PPI users in our study, particularly among Korean adults aged 40 years or older. Conversely, the protective effects of prolonged PPI use that was observed in certain subgroups may be linked to several beneficial properties of PPIs. Interestingly, PPI use seemed to tend to be associated with decreased non-gastrointestinal cancer risks of breast, cervical, endometrial, and ovarian cancers in specific demographic groups [44], which may be consistent with a protective effect among certain demographic subgroups in our study.

This study has several strengths. First, it is based on a large, nationwide cohort with comprehensive healthcare data, ensuring a high degree of generalizability. The use of propensity score overlap weighting allowed for robust adjustment of confounding factors, providing more reliable estimates of the connections between PPI usage and lung cancer outcomes. The thoroughly adjusted consideration of socioeconomic status, along with risk factors and comorbidities that are potentially linked to lung cancer or PPI usage [14,45] (e.g., pressure levels, total cholesterol, fasting glycemic values, smoking habits, obesity, and alcohol intake), may represent a significant strength of this study. Furthermore, our study controlled for other acid suppressors, such as H2 receptor antagonists, minimizing potential confounding effects.

Despite its strengths, this study has several limitations that should be acknowledged. First, the retrospective design precludes causal inferences, and we relied on administrative claims data, which may lack detailed clinical information, such as family history, genetic factors, and adherence to prescribed medication. Second, our exclusion criteria, which limited the analysis to lung cancer patients who underwent surgery, chemotherapy, or radiotherapy, may have resulted in the under-representation of older or poor-performing patients with advanced lung cancer. This could have influenced the observed association, particularly in older subgroups. Third, the use of ICD codes to identify cases and comorbidities may introduce coding errors or misclassification bias. Lastly, the findings are based on a Korean population and may not be generalizable to other ethnic or geographical groups.

## 5. Conclusions

This study highlights the complex relationship between PPI use and lung cancer outcomes. While prolonged PPI use was associated with a reduced risk of lung cancer in certain subgroups, such as older adults and GERD patients, it was also correlated with increased mortality in high-risk populations, including smokers, underweight individuals, and those with elevated cholesterol. These findings underscore the importance of careful evaluation of patient-specific factors, particularly when prescribing PPIs for long-term use. Further research is warranted to confirm these findings and refine risk stratification for safer clinical decision-making.

## Figures and Tables

**Table 1:** Crude and overlap propensity score-weighted odds ratios of the history and duration of PPI use for incident lung cancer.

Characteristics	Lung Cancer	Control	Odds Ratios (95% Confidence Intervals)			
	(Exposure/Total, %)	(Exposure/Total, %)	Crude	*p*	Adjusted †	*p*
History of PPI use						
Non-user	5450/6795 (80.21)	23,879/27,180 (87.86)	1		1	
User	1345/6795 (19.79)	3301/27,180 (12.14)	1.79 (1.66–1.91)	<0.001 *	1.19 (1.12–1.26)	<0.001 *
Duration of PPI use						
<30 days	6224/6795 (91.60)	25,541/27,180 (93.97)	1		1	
≥30 days	571/6795 (8.40)	1639/27,180 (6.03)	1.43 (1.29–1.58)	<0.001 *	0.87 (0.80–0.94)	<0.001 *

Abbreviations: PPI, proton pump inhibitor. *: logistic regression model, significance at *p* < 0.05. †: adjusted using overlap propensity score weighting for age, sex, income status, region of residence, systolic blood pressure, diastolic blood pressure, fasting blood glucose, total cholesterol, weight status, smoking status, alcohol consumption, Charlson Comorbidity Index score, number of gastroesophageal reflux disease (GERD) treatments, and duration of treatment with H2 receptor antagonist.

**Table 2:** General characteristics of participants with lung cancer.

Characteristics	Before Overlap Weighting Adjustment			After Overlap Weighting Adjustment		
	Dead Participants	Survived Participants	Standardized Difference	Dead Participants	Survived Participants	Standardized Difference
Age (years; n, %)			0.26			0.00
40–44	14 (0.33)	5 (0.20)		3 (0.23)	3 (0.23)	
45–49	82 (1.93)	31 (1.22)		20 (1.65)	20 (1.65)	
50–54	235 (5.52)	147 (5.79)		63 (5.24)	63 (5.24)	
55–59	384 (9.02)	373 (14.70)		135 (11.21)	135 (11.21)	
60–64	664 (15.60)	514 (20.25)		213 (17.74)	213 (17.74)	
65–69	934 (21.94)	515 (20.29)		258 (21.47)	258 (21.47)	
70–74	1002 (23.54)	446 (17.57)		243 (20.22)	243 (20.22)	
75–79	638 (14.99)	369 (14.54)		188 (15.61)	188 (15.61)	
80–84	248 (5.83)	118 (4.65)		66 (5.51)	66 (5.51)	
85+	56 (1.32)	20 (0.79)		14 (1.12)	14 (1.12)	
Sex (n, %)			0.37			0.00
Male	3521 (82.71)	1702 (67.06)		911 (75.75)	911 (75.75)	
Female	736 (17.29)	836 (32.94)		292 (24.25)	292 (24.25)	
Income (n, %)			0.17			0.00
1 (lowest)	656 (15.41)	341 (13.44)		174 (14.47)	174 (14.47)	
2	573 (13.46)	229 (9.02)		127 (10.56)	127 (10.56)	
3	744 (17.48)	404 (15.92)		202 (16.82)	202 (16.82)	
4	946 (22.22)	572 (22.54)		274 (22.75)	274 (22.75)	
5 (highest)	1338 (31.43)	992 (39.09)		426 (35.40)	426 (35.40)	
Region of residence (n, %)			0.05			0.00
Urban	1741 (40.90)	1104 (43.50)		509 (42.33)	509 (42.33)	
Rural	2516 (59.10)	1434 (56.50)		694 (57.67)	694 (57.67)	
Weight status † (n, %)			0.15			0.00
Underweight	216 (5.07)	65 (2.56)		41 (3.44)	41 (3.44)	
Normal	1799 (42.26)	959 (37.79)		470 (39.09)	470 (39.09)	
Overweight	1048 (24.62)	691 (27.23)		317 (26.33)	317 (26.33)	
Obese I	1100 (25.84)	762 (30.02)		347 (28.88)	347 (28.88)	
Obese II	94 (2.21)	61 (2.40)		27 (2.27)	27 (2.27)	
Smoking status (n, %)			0.32			0.00
Nonsmoker	1674 (39.32)	1244 (49.01)		515 (42.83)	515 (42.83)	
Past smoker	900 (21.14)	659 (25.97)		304 (25.24)	304 (25.24)	
Current smoker	1683 (39.53)	635 (25.02)		384 (31.94)	384 (31.94)	
Alcohol consumption (n, %)			0.06			0.00
<1 time a week	2528 (59.38)	1578 (62.17)		730 (60.68)	730 (60.68)	
≥1 time a week	1729 (40.62)	960 (37.83)		473 (39.32)	473 (39.32)	
SBP (mmHg;mean, SD)	128.57(17.16)	126.48 (15.24)	0.13	127.58 (8.83)	127.58 (10.78)	0.00
DBP (mmHg;mean, SD)	78.00 (10.50)	76.93 (9.86)	0.11	77.42 (5.43)	77.42 (6.86)	0.00
Fasting blood glucose (mg/dL;mean, SD)	104.41(30.50)	103.24 (24.55)	0.04	104.10 (15.42)	104.10 (17.90)	0.00
Total cholesterol (mg/dL;mean, SD)	189.27 (38.04)	190.51 (38.91)	0.03	189.39 (20.18)	189.39 (26.83)	0.00
CCI score (Mean, SD)	5.61 (2.08)	3.75 (1.98)	0.92	4.53 (1.09)	4.53 (1.48)	0.00
No. of GERD treatments (mean, SD)	0.78 (2.44)	0.99 (2.29)	0.09	0.93 (1.52)	0.93 (1.53)	0.00
No. of treatments for H2RA (mean, SD)	6.27 (22.02)	2.78 (14.91)	0.19	3.83 (6.28)	3.83 (13.88)	0.00
History of PPI use (n, %)			0.13			0.12
Non-user	3335 (78.34)	2115 (83.33)		947 (78.73)	1000 (83.15)	
User	922 (21.66)	423 (16.67)		256 (21.27)	203 (16.85)	
Status based on duration of PPI use (n, %)			0.06			0.06
<30 days	3873 (90.98)	2351 (92.63)		1098 (91.25)	1117 (92.84)	
≥30 days	384 (9.02)	187 (7.37)		105 (8.75)	86 (7.16)	

Abbreviations: CCI, Charlson Comorbidity Index; SBP, systolic blood pressure; DBP, diastolic blood pressure; SD, standard deviation; H2RA, H2 receptor antagonist; GERD, gastroesophageal reflux disease. † Weight status (body mass index (BMI), kg/m^2^) was categorized as <18.5 (underweight), ≥18.5 to <23 (normal), ≥23 to <25 (overweight), ≥25 to <30 (obese I), and ≥30 (obese II).

**Table 3:** Crude and overlap propensity score weighted odd ratios of the history and duration of PPI use for mortality in lung cancer participants.

Characteristics	Dead pts	Survived pts	Odds Ratios (95% Confidence Intervals)			
	(Exposure/Total, %)	(Exposure/Total, %)	Crude	*p*	Adjusted Model with OW †	*p*
History of PPI use						
Non-user	3335/4257 (78.34)	2115/2538 (83.33)	1		1	
User	922/4257 (21.66)	423/2538 (16.67)	1.38 (1.22–1.57)	<0.001 *	1.36 (1.20–1.55)	<0.001 *
Duration of PPI use						
<30 days	3873/4257 (90.98)	2351/2538 (92.63)	1		1	
≥30 days	384/4257 (9.02)	187/2538 (7.37)	1.25 (1.04–1.50)	0.018 *	1.27 (1.05–1.53)	0.012 *

Abbreviations: PPI, proton pump inhibitor; pts, patients; GERD, gastroesophageal reflux disease; OW, overlap propensity score-weighted adjustment. * Logistic regression model, Significance at *p* < 0.05. † Adjusted for age, sex, income status, region of residence, systolic blood pressure, diastolic blood pressure, fasting blood glucose, total cholesterol, weight status, smoking status, alcohol consumption, Charlson Comorbidity Index scores, number of GERD treatments, and duration of treatment with H2-receptor antagonist.

## Data Availability

All data were obtained from the database of the National Health Insurance Sharing Service (NHISS) and are available at https://nhiss.nhis.or.kr/ (accessed on 10 April 2024). The NHISS allows access to all data (downloaded from the website) for any researcher who agrees to follow the research ethics and pays a processing fee.

## Supplementary Materials

The following are available online at https://www.mdpi.com/article/10.3390/cancers17050877/s1: Table S1: Baseline characteristics of participants before and after propensity score overlap weighting adjustment; Table S2: Subgroup analysis of crude and overlap propensity score-weighted odds ratios for PPI use history and lung cancer; Table S3: Subgroup analysis of crude and adjusted odds ratios for the duration of PPI use and lung cancer; Table S4: Subgroup analysis of crude and overlap propensity score-weighted odds ratios for PPI use history and mortality in lung cancer participants; Table S5: Subgroup analysis of crude and overlap propensity score-weighted odds ratios for the duration of PPI use and mortality in lung cancer participants.
