# Supplementary material for: Proton Pump Inhibitor Use and Its Association with Lung Cancer Likelihood and Mortality: A Nationwide Nested Case–Control Study in Korea
Source: Cancers (Basel). 2025 Mar 4;17(5):877. doi: 10.3390/cancers17050877 (PMC11899281; doi:10.3390/cancers17050877)
Supplement: Supplementary file 1 [file cancers-17-00877-s001.zip › cancers-3442219-supplementary.pdf]

Supplementary Table S1. Baseline characteristics of participants before and after propensity score overlap weighting adjustment.

Characteristics	Before Overlap weighting adjustment			After Overlap weighting adjustment		
	Lung cancer	Control	Standardized Difference	Lung cancer	Control	Standardized Difference
Age (years;n, %)			0.00			0.00
40–44	19 (0.28)	76 (0.28)		5 (0.15)	5 (0.15)	
45–49	113 (1.66)	452 (1.66)		41 (1.30)	41 (1.30)	
50–54	382 (5.62)	1528 (5.62)		142 (4.54)	142 (4.54)	
55–59	757 (11.14)	3028 (11.14)		301 (9.61)	301 (9.61)	
60–64	1178 (17.34)	4712 (17.34)		504 (16.12)	504 (16.12)	
65–69	1449 (21.32)	5796 (21.32)		651 (20.80)	651 (20.80)	
70–74	1448 (21.31)	5792 (21.31)		704 (22.49)	704 (22.49)	
75–79	1007 (14.82)	4028 (14.82)		529 (16.92)	529 (16.92)	
80–84	366 (5.39)	1464 (5.39)		209 (6.69)	209 (6.69)	
85+	76 (1.12)	304 (1.12)		43 (1.38)	43 (1.38)	
Sex (n, %)			0.00			0.00
Male	5223 (76.87)	20,892 (76.87)		2409 (77.02)	2409 (77.03)	
Female	1572 (23.13)	6288 (23.13)		719 (22.98)	719 (22.97)	
Income (n, %)			0.00			0.00
1 (lowest)	997 (14.67)	3988 (14.67)		476 (15.22)	476 (15.22)	
2	802 (11.80)	3208 (11.80)		355 (11.36)	355 (11.36)	
3	1148 (16.89)	4592 (16.89)		516 (16.50)	516 (16.50)	
4	1518 (22.34)	6072 (22.34)		690 (22.05)	690 (22.05)	
5 (highest)	2330 (34.29)	9320 (34.29)		1091 (34.87)	1091 (34.87)	
Region of residence (n, %)			0.00			0.00
Urban	2845 (41.87)	11,380 (41.87)		1268 (40.56)	1269 (40.56)	
Rural	3950 (58.13)	15,800 (58.13)		1859 (59.44)	1859 (59.44)	
Weight status† (n, %)			0.15			0.00
Underweight	281 (4.14)	809 (2.98)		124 (3.96)	124 (3.96)	
Normal	2758 (40.59)	9517 (35.01)		1210 (38.69)	1210 (38.69)	
Overweight	1739 (25.59)	7642 (28.12)		829 (26.50)	829 (26.50)	
Obese I	1862 (27.40)	8532 (31.39)		893 (28.55)	893 (28.55)	
Obese II	155 (2.28)	680 (2.50)		72 (2.31)	72 (2.31)	
Smoking status (n, %)			0.37			0.00
Nonsmoker	2918 (42.94)	15,713 (57.81)		1498 (47.88)	1498 (47.88)	
Past smoker	1559 (22.94)	6129 (22.55)		749 (23.93)	749 (23.93)	
Current smoker	2318 (34.11)	5338 (19.64)		882 (28.19)	882 (28.19)	
Alcohol consumption (n, %)			0.03			0.00
<1 time a week	4106 (60.43)	16,879 (62.10)		1945 (62.19)	1945 (62.19)	
≥1 time a week	2689 (39.57)	10,301 (37.90)		1183 (37.81)	1183 (37.81)	
SBP (mmHg;mean, SD)	127.79 (16.50)	128.68 (16.32)	0.05	128.42 (11.06)	128.42 (5.68)	0.00
DBP (mmHg;mean, SD)	77.60 (10.28)	78.45 (10.42)	0.08	77.90 (6.92)	77.90 (3.62)	0.00

Fasting blood glucose (mg/dL;mean, SD)	103.97 (28.43)	104.12 (30.35)		104.63 (19.44)	104.63 (10.36)	
Total cholesterol level (mg/dL;mean, SD)	189.73 (38.37)	192.67 (38.64)	0.08	189.70 (26.05)	189.70 (13.52)	0.00
CCI score (mean, SD)	4.92 (2.23)	1.15 (1.76)	0.14	3.52 (1.18)	3.52 (0.93)	0.00
No. of GERD treatments (mean, SD)	0.86 (2.39)	0.60 (2.18)	0.11	0.81 (1.48)	0.81 (1.05)	0.00
No. of treatments for H2RA (mean, SD)	4.97 (19.74)	3.11 (19.35)	0.09	4.83 (14.81)	4.83 (8.11)	0.00
History of PPI use (n, %)			0.21			0.06
Non-user	5450 (80.21)	23,879 (87.86)		2572 (82.22)	2640 (84.41)	
User	1345 (19.79)	3301 (12.14)		556 (17.78)	488 (15.59)	
Status based on duration of PPI use (n, %)			0.09			0.04
<30 days	6224 (91.60)	25,541 (93.97)		2894 (92.52)	2864 (91.56)	
≥ 30 days	571 (8.40)	1639 (6.03)		234 (7.48)	264 (8.44)	

Abbreviations: CCI, Charlson Comorbidity Index; SBP, Systolic blood pressure; DBP, Diastolic blood pressure; SD, standard deviation; H2RA, H2-receptor antagonist; GERD, gastroesophageal reflux disease. †Weight status (body mass index (BMI), kg/m2) was categorized as <18.5 (underweight), ≥18.5 to <23 (normal), ≥23 to <25 (overweight), ≥25 to <30 (obese I), and ≥30 (obese II).

Supplementary Table S2. Subgroup analysis of crude and overlap propensity score-weighted odds ratios for PPI use history and lung cancer

Characteristics	Lung cancer (exposure/total, %)	Control (exposure/total, %)	Odd ratios for lung cancer (95% confidence interval) Crude	P-value	Overlap weighted model †	P-value
Age < 70 years old (n = 19,490)						
Non-user	3,144/3,898 (80.66)	13,776/15,592 (88.35)	1		1	
User	754/3,898 (19.34)	1,816/15,592 (11.65)	1.82 (1.66-2.00)	<0.001*	1.27 (1.17-1.38)	<0.001*
Age ≥ 70 years old (n = 14,485)						
Non-user	2,306/2,897(79.60)	10,103/11,588 (87.19)	1		1	
User	591/2,897 (20.40)	1,485/11,588 (12.81)	1.74 (1.57-1.94)	<0.001*	1.13 (1.04-1.24)	0.007*
Men (n = 26,115)						
Non-user	4,212/5,223 (80.64)	18,427/20,892(88.20)	1		1	
User	1,011/5,223 (19.36)	2,465/20,892(11.80)	1.79 (1.66-1.94)	<0.001*	1.21 (1.13-1.30)	<0.001*
Women (n =7,860)						
Non-user	1,238/1,572 (78.75)	5,452/6,288 (86.70)	1		1	
User	334/1,572 (21.25)	836/6,288 (13.30)	1.76 (1.53-2.03)	<0.001*	1.07 (0.94-1.21)	0.304
Low income (n = 14,735)						
Non-user	2,338/2,947 (79.33)	10,334/11,788 (87.67)	1		1	
User	609/2,947 (20.67)	1,454/11,788 (12.33)	1.85 (1.67-2.06)	<0.001*	1.16 (1.06-1.26)	0.002*
High income (n = 19,240)						
Non-user	3,112/3,848 (80.87)	13,545/15,392(88.00)	1		1	
User	736/3,848 (19.13)	1,847/15,392(12.00)	1.73 (1.58-1.91)	<0.001*	1.22 (1.13-1.32)	<0.001*
Urban residents (n = 14,225)						
Non-user	2,318/2,845 (81.48)	10,093/11,380 (88.69)	1		1	
User	527/2,845 (18.52)	1,287/11,380 (11.31)	1.78 (1.60-1.99)	<0.001*	1.16 (1.06-1.28)	0.002*
Rural residents (n = 19,750)						
Non-user	3,132/3,950 (79.29)	13,786/15,800 (87.25)	1		1	
User	818/3,950 (20.71)	2,014/15,800 (12.75)	1.79 (1.63-1.96)	<0.001*	1.20 (1.11-1.30)	<0.001*
Underweight (n = 1,090)						
Non-user	214/281 (76.16)	709/809 (87.64)	1		1	
User	67/281 (23.84)	100/809 (12.36)	2.22 (1.57-3.13)	<0.001*	1.68 (1.18-2.39)	0.004*
Normal weight (n = 12,275)						
Non-user	2,215/2,758 (80.31)	8,347/9,517 (87.71)	1		1	
User	543/2,758 (19.69)	1,170/9,517 (12.29)	1.75 (1.56-1.96)	<0.001*	1.15 (1.04-1.26)	0.007*
Overweight (n = 9,381)						
Non-user	1,387/1,739 (79.76)	6,719/7,642 (87.92)	1		1	

User	352/1,739 (20.24)	923/7,642 (12.08)	1.85 (1.61-2.12)	<0.001*	1.30 (1.16-1.45)	<0.001*
Obese (n = 11,229)						
Non-user	1,634/2,017 (81.01)	8,104/9,212 (87.97)	1		1	
User	383/2,017 (18.99)	1,108/9,212 (12.03)	1.71 (1.51-1.95)	<0.001*	1.08 (0.97-1.20)	0.142
Non-smoker (n = 18,631)						
Non-user	2,324/2,918 (79.64)	13,808/15,713 (87.88)	1		1	
User	594/2,918 (20.36)	1,905/15,713 (12.12)	1.85 (1.67-2.05)	<0.001*	1.26 (1.16-1.37)	<0.001*
Past smoker and current smoker (n = 15,344)						
Non-user	3,126/3,877 (80.63)	10,071/11,467 (87.83)	1		1	
User	751/3,877 (19.37)	1,396/11,467 (12.17)	1.73 (1.57-1.91)	<0.001*	1.12 (1.02-1.22)	0.015*
Alcohol consumption < 1 time a week (n = 20,985)						
Non-user	3,273/4,106 (79.71)	14,776/16,879 (87.54)	1		1	
User	833/4,106 (20.29)	2,103/16,879 (12.46)	1.79 (1.64-1.95)	<0.001*	1.16 (1.08-1.25)	<0.001*
Alcohol consumption ≥ 1 time a week (n = 12,990)						
Non-user	2,177/2,689 (80.96)	9,103/10,301 (88.37)	1		1	
User	512/2,689 (19.04)	1,198/10,301 (11.63)	1.79 (1.60-2.00)	<0.001*	1.23 (1.12-1.36)	<0.001*
SBP < 140 mmHg and DBP < 90 mmHg (n = 8,100)						
Non-user	1,373/1,758 (78.10)	5,550/6,342 (87.51)	1		1	
User	385/1,758 (21.90)	792/6,342 (12.49)	1.96 (1.72-2.25)	<0.001*	1.33 (1.18-1.51)	<0.001*
SBP ≥ 140 mmHg or DBP ≥ 90 mmHg (n = 25,875)						
Non-user	4,077/5,037 (80.94)	18,329/20,838 (87.96)	1		1	
User	960/5,037 (19.06)	2,509/20,838 (12.04)	1.72 (1.59-1.87)	<0.001*	1.14 (1.06-1.22)	<0.001*
Fasting blood glucose < 100 mg/dL (n = 18,760)						
Non-user	2,964/3,700 (80.11)	13,204/15,060 (87.68)	1		1	
User	736/3,700 (19.89)	1,856/15,060 (12.32)	1.77 (1.61-1.94)	<0.001*	1.22 (1.13-1.33)	<0.001*
Fasting blood glucose ≥ 100 mg/dL (n = 15,215)						
Non-user	2,486/3,095 (80.32)	10,675/12,120 (88.08)	1		1	
User	609/3,095 (19.68)	1,445/12,120 (11.92)	1.81 (1.63-2.01)	<0.001*	1.15 (1.05-1.25)	0.003*
Total cholesterol < 200mg/dL (n = 20,380)						
Non-user	3,380/4,225 (80.00)	14,104/16,155 (87.30)	1		1	
User	845/4,225 (20.00)	2,051/16,155 (12.70)	1.72 (1.57-1.88)	<0.001*	1.18 (1.09-1.27)	<0.001*
Total cholesterol ≥ 200mg/dL (n = 13,595)						
Non-user	2,070/2,570 (80.54)	9,775/11,025 (88.66)	1		1	
User	500/2,570 (19.46)	1,250/11,025 (11.34)	1.89 (1.69-2.12)	<0.001*	1.22 (1.11-1.35)	<0.001*
CCI scores = 0 (n =14,623)						

Non-user	21/21 (100.00)	13,086/14,602 (89.62)	1		1	
User	0/21 (0.00)	1,516/14,602 (10.38)	N/A		N/A	
CCI score = 1 (n = 4,999)						
Non-user	5/5 (100.00)	4,360/4,994 (87.30)	1		1	
User	0/5 (0.00)	634/4,994 (12.70)	N/A		N/A	
CCI score ≥ 2 (n = 14,353)						
Non-user	5,424/6,769 (80.13)	6,433/7,584 (84.82)	1		1	
User	1,345/6,769 (19.87)	1,151/7,584 (15.18)	1.39 (1.27-1.51)	<0.001*	1.16 (1.05-1.27)	0.003*
Non-GERD user (n = 27,422)						
Non-user	4,508/5,108 (88.25)	21,085/22,314 (94.49)				
User	600/5,108 (11.75)	1,229/22,314 (5.51)	2.28 (2.06-2.53)	<0.001*	1.51 (1.39-1.64)	<0.001*
GERD user (n = 6,553)						
Non-user	942/1,687 (55.84)	2,794/4,866 (57.42)				
User	745/1,687 (44.16)	2,072/4,866 (42.58)	1.07 (0.95-1.19)	0.259	0.65 (0.59-0.72)	<0.001*
H2 blocker user (n = 5,502)						
Non-user	1,407/1,983 (70.95)	2,709/3,519 (76.98)				
User	576/1,983 (29.05)	810/3,519 (23.02)	1.37 (1.21-1.55)	<0.001*	1.13 (1.00-1.27)	0.057

Abbreviations: CCI, Charlson Comorbidity Index; SBP, Systolic blood pressure; DBP, Diastolic blood pressure; PPI, proton pump inhibitor; GERD, gastroesophageal reflux disease

* Significance at P < 0.05

† Adjusted for age, sex, income, region of residence, obesity, smoking, alcohol consumption, SBP, DBP, fasting blood glucose, total cholesterol, CCI scores, GERD and H2-receptor antagonist.

Supplementary Table S3. Subgroup analysis of crude and adjusted odds ratios for the duration of PPI use and lung cancer.

Characteristics	Lung cancer (exposure/total, %)	Control (exposure/total, %)	Odd ratios for Lung cancer (95% confidence interval)			
			Crude	P-value	Overlap weighted model †	P-value
Age < 70 years old (n = 19,490)						
< 30 days	3,599/3,898 (92.33)	14,736/15,592 (94.51)	1		1	
≥ 30 days	299/3,898 (7.67)	856/15,592 (5.49)	1.43 (1.25-1.64)	<0.001*	0.91 (0.81-1.02)	0.114
Age ≥ 70 years old (n = 14,485)						
< 30 days	2,625/2,897 (90.61)	10,805/11,588 (93.24)	1		1	
≥ 30 days	272/2,897 (9.39)	783/11,588 (6.76)	1.43 (1.24-1.65)	<0.001*	0.83 (0.74-0.94)	0.004*
Men (n = 26,115)						
< 30 days	4,782/5,223 (91.56)	19,670/20,892 (94.15)	1		1	
≥ 30 days	441/5,223 (8.44)	1,222/20,892 (5.85)	1.48 (1.33-1.66)	<0.001*	0.89 (0.81-0.98)	0.017*
Women (n =7,860)						
< 30 days	1,442/1,572 (91.73)	5,871/6,288 (93.37)	1		1	
≥ 30 days	130/1,572 (8.27)	417/6,288 (6.63)	1.27 (1.03-1.56)	0.023*	0.76 (0.64-0.90)	0.002*
Low income (n = 14,735)						
< 30 days	2,690/2,947 (91.28)	11,070/11,788 (93.91)	1		1	
≥ 30 days	257/2,947 (8.72)	718/11,788 (6.09)	1.47 (1.27-1.71)	<0.001*	0.84 (0.74-0.95)	0.005*
High income (n = 19,240)						
< 30 days	3,534/3,848 (91.84)	14,471/15,392 (94.02)	1		1	
≥ 30 days	314/3,848 (8.16)	921/15,392 (5.98)	1.40 (1.22-1.60)	<0.001*	0.89 (0.80-1.00)	0.041*
Urban residents (n = 14,225)						
< 30 days	2,638/2,845 (92.72)	10,754/11,380 (94.50)	1		1	
≥ 30 days	207/2,845 (7.28)	626/11,380 (5.50)	1.35 (1.15-1.59)	<0.001*	0.81 (0.71-0.92)	0.002*
Rural residents (n = 19,750)						
< 30 days	3,586/3,950 (90.78)	14,787/15,800 (93.59)	1		1	
≥ 30 days	364/3,950 (9.22)	1,013/15,800 (6.41)	1.48 (1.31-1.68)	<0.001*	0.90 (0.81-1.00)	0.043*
Underweight (n = 1,090)						
< 30 days	251/281 (89.32)	763/809 (94.31)	1		1	
≥ 30 days	30/281 (10.68)	46/809 (5.69)	1.98 (1.22-3.21)	0.005*	1.16 (0.72-1.88)	0.538
Normal weight (n = 12,275)						
< 30 days	2,540/2,758 (92.10)	8,947/9,517 (94.01)	1		1	
≥ 30 days	218/2,758 (7.90)	570/9,517 (5.99)	1.35 (1.15-1.58)	<0.001*	0.77 (0.67-0.89)	<0.001*
Overweight (n = 9,381)						
< 30 days	1,601/1,739 (92.06)	7,184/7,642 (94.01)	1		1	

≥ 30 days	138/1,739 (7.94)	458/7,642 (5.99)	1.35 (1.11-1.65)	0.003*	0.98 (0.84-1.15)	0.827
Obese (n = 11,229)						
< 30 days	1,832/2,017 (90.83)	8,647/9,212 (93.87)	1		1	
≥ 30 days	185/2,017 (9.17)	565/9,212 (6.13)	1.55 (1.30-1.84)	<0.001*	0.84 (0.73-0.96)	0.012*
Non-smoker (n = 18,631)						
< 30 days	2,671/2,918 (91.54)	14,756/15,713 (93.91)	1		1	
≥ 30 days	247/2,918 (8.46)	957/15,713 (6.09)	1.43 (1.23-1.65)	<0.001*	0.87 (0.78-0.97)	0.013*
Past smoker and current smoker (n = 15,344)						
< 30 days	3,553/3,877 (91.64)	10,785/11,467 (94.05)	1		1	
≥ 30 days	324/3,877 (8.36)	682/11,467 (5.95)	1.44 (1.26-1.65)	<0.001*	0.85 (0.75-0.96)	0.010*
Alcohol consumption < 1 time a week (n = 20,985)						
< 30 days	3,738/4,106 (91.04)	15,809/16,879 (93.66)	1		1	
≥ 30 days	368/4,106 (8.96)	1,070/16,879 (6.34)	1.46 (1.29-1.65)	<0.001*	0.85 (0.77-0.94)	0.002*
Alcohol consumption ≥ 1 time a week (n = 12,990)						
< 30 days	2,486/2,689 (92.45)	9,732/10,301 (94.48)	1		1	
≥ 30 days	203/2,689 (7.55)	569/10,301 (5.52)	1.40 (1.18-1.65)	<0.001*	0.90 (0.78-1.04)	0.157
SBP < 140 mmHg and DBP < 90 mmHg (n = 8,100)						
< 30 days	1,596/1,758 (90.78)	5,953/6,342 (93.87)	1		1	
≥ 30 days	162/1,758 (9.22)	389/6,342 (6.13)	1.55 (1.28-1.88)	<0.001*	0.97 (0.82-1.14)	0.680
SBP ≥ 140 mmHg or DBP ≥ 90 mmHg (n = 25,875)						
< 30 days	4,628/5,037 (91.88)	19,588/20,838 (94.00)	1		1	
≥ 30 days	409/5,037 (8.12)	1,250/20,838 (6.00)	1.39 (1.23-1.56)	<0.001*	0.83 (0.75-0.91)	<0.001*
Fasting blood glucose < 100 mg/dL (n = 18,760)						
< 30 days	3,401/3,700 (91.92)	14,156/15,060 (94.00)	1		1	
≥ 30 days	299/3,700 (8.08)	904/15,060 (6.00)	1.38 (1.20-1.58)	<0.001*	0.89 (0.80-1.00)	0.047*
Fasting blood glucose ≥ 100 mg/dL (n = 15,215)						
< 30 days	2,823/3,095 (91.21)	11,385/12,120 (93.94)	1		1	
≥ 30 days	272/3,095 (8.79)	735/12,120 (6.06)	1.49 (1.29-1.73)	<0.001*	0.84 (0.74-0.95)	0.004*
Total cholesterol < 200mg/dL (n = 20,380)						
< 30 days	3,857/4,225 (91.29)	15,092/16,155 (93.42)	1		1	
≥ 30 days	368/4,225 (8.71)	1,063/16,155 (6.58)	1.35 (1.20-1.53)	<0.001*	0.84 (0.76-0.94)	0.001*
Total cholesterol ≥ 200mg/dL (n = 13,595)						
< 30 days	2,367/2,570 (92.10)	10,449/11,025 (94.78)	1		1	
≥ 30 days	203/2,570 (7.90)	576/11,025 (5.22)	1.56 (1.32-1.84)	<0.001*	0.92 (0.80-1.05)	0.204
CCI scores = 0 (n =14,623)						

< 30 days	21/21 (100.00)	13,904/14,602 (95.22)	1		1	
≥ 30 days	0/21 (0.00)	698/14,602 (4.78)	N/A		N/A	
CCI score = 1 (n = 4,999)						
< 30 days	5/5 (100.00)	4,669/4,994 (93.49)	1		1	
≥ 30 days	0/5 (0.00)	325/4,994 (6.51)	N/A		N/A	
CCI score ≥ 2 (n = 14,353)						
< 30 days	6,198/6,769 (91.56)	6,968/7,584 (91.88)	1		1	
≥ 30 days	571/6,769 (8.44)	616/7,584 (8.12)	1.04 (0.93-1.17)	0.496	0.84 (0.73-0.95)	0.008*
Non-GERD user (n = 27,422)						
< 30 days	4,904/5,108 (96.01)	21,788/22,314 (97.64)	1		1	
≥ 30 days	204/5,108 (3.99)	526/22,314 (2.36)	1.72 (1.46-2.03)	<0.001*	1.12 (0.98-1.27)	0.086
GERD user (n = 6,553)						
< 30 days	1,320/1,687 (78.25)	3,753/4,866 (77.13)	1		1	
≥ 30 days	367/1,687 (21.75)	1,113/4,866 (22.87)	0.94 (0.82-1.07)	0.344	0.55 (0.49-0.62)	<0.001*
Non-H2 blocker ≥ 30 days (n = 28,473)						
< 30 days	4,485/4,812 (93.20)	22,434/23,661 (94.81)	1		1	
≥ 30 days	327/4,812 (6.80)	1,227/23,661 (5.19)	1.33 (1.18-1.51)	<0.001*	0.77 (0.70-0.85)	<0.001*
H2 blocker ≥ 30 days (n = 5,502)						
< 30 days	1,739/1,983 (87.70)	3,107/3,519 (88.29)	1		1	
≥ 30 days	244/1,983 (12.30)	412/3,519 (11.71)	1.06 (0.89-1.25)	0.510	0.91 (0.77-1.07)	0.241

Abbreviations: CCI, Charlson Comorbidity Index; SBP, Systolic blood pressure; DBP, Diastolic blood pressure; PPI, proton pump inhibitor; GERD, gastroesophageal reflux disease.

* Significance at P < 0.05

† Adjusted for age, sex, income, region of residence, SBP, DBP, fasting blood glucose, total cholesterol, obesity, smoking, alcohol consumption, CCI scores, GERD and H2-receptor antagonist.

Supplementary Table S4. Subgroup analysis of crude and overlap propensity score-weighted odds ratios for PPI use history and mortality in lung cancer participants.

Characteristics	Dead participants (exposure/total, %)	Survived participants (exposure/total, %)	Odd ratios for mortality (95% confidence interval)			
			Crude	P-value	Overlap weighted model †	P-value
Age < 70 years old (n = 3,898)						
Non-user	1,844/2,313 (79.72)	1,300/1,585 (82.02)	1		1	
User	469/2,313 (20.28)	285/1,585 (17.98)	1.16 (0.99-1.37)	0.075	1.15 (0.97-1.36)	0.109
Age ≥ 70 years old (n = 2,897)						
Non-user	1,491/1,944 (76.70)	815/953 (85.52)	1		1	
User	453/1,944 (23.30)	138/953 (14.48)	1.79 (1.46-2.21)	<0.001*	1.77 (1.45-2.16)	<0.001*
Men (n = 5,223)						
Non-user	2,782/3,521 (79.01)	1,430/1,702 (84.02)	1		1	
User	739/3,521 (20.99)	272/1,702 (15.98)	1.40 (1.20-1.63)	<0.001*	1.37 (1.19-1.59)	<0.001*
Women (n = 1,572)						
Non-user	553/736 (75.14)	685/836 (81.94)	1		1	
User	183/736 (24.86)	151/836 (18.06)	1.50 (1.18-1.91)	0.001*	1.32 (1.02-1.71)	0.036*
Low income (n = 2,947)						
Non-user	1,534/1,973 (77.75)	804/974 (82.55)	1		1	
User	439/1,973 (22.25)	170/974 (17.45)	1.35 (1.11-1.65)	0.003*	1.38 (1.14-1.67)	0.001*
High income (n = 3,848)						
Non-user	1,801/2,284 (78.85)	1,311/1,564 (83.82)	1		1	
User	483/2,284 (21.15)	253/1,564 (16.18)	1.39 (1.17-1.64)	<0.001*	1.36 (1.15-1.61)	<0.001*
Urban residents (n = 2,845)						
Non-user	1,382/1,741 (79.38)	936/1,104 (84.78)	1		1	
User	359/1,741 (20.62)	168/1,104 (15.22)	1.45 (1.18-1.77)	<0.001*	1.46 (1.19-1.79)	<0.001*
Rural residents (n = 3,950)						
Non-user	1,953/2,516 (77.62)	1,179/1,434 (82.22)	1		1	
User	563/2,516 (22.38)	255/1,434 (17.78)	1.33 (1.13-1.57)	0.001*	1.31 (1.11-1.54)	0.001*
Underweight (n = 281)						
Non-user	157/216 (72.69)	57/65 (87.69)	1		1	
User	59/216 (27.31)	8/65 (12.31)	2.68 (1.21-5.95)	0.016*	3.76 (1.78-7.97)	0.001*
Normal weight (n = 2,758)						
Non-user	1,406/1,799 (78.15)	809/959 (84.36)	1		1	
User	393/1,799 (21.85)	150/959 (15.64)	1.51 (1.23-1.85)	<0.001*	1.46 (1.19-1.78)	<0.001*
Overweight (n = 1,739)						
Non-user	817/1,048 (77.96)	570/691 (82.49)	1		1	

User	231/1,048 (22.04)	121/691 (17.51)	1.33 (1.04-1.70)	0.022*	1.42 (1.10-1.82)	0.007*
Obese (n = 2,017)						
Non-user	955/1,194 (79.98)	679/823 (82.50)	1		1	
User	239/1,194 (20.02)	144/823 (17.50)	1.18 (0.94-1.48)	0.156	1.14 (0.90-1.45)	0.263
Non-smoker (n = 2,918)						
Non-user	1,309/1,674 (78.20)	1,015/1,244 (81.59)	1		1	
User	365/1,674 (21.80)	229/1,244 (18.41)	1.24 (1.03-1.49)	0.024*	1.13 (0.94-1.37)	0.190
Past smoker and current smoker (n = 3,877)						
Non-user	2,026/2,583 (78.44)	1,100/1,294 (85.01)	1		1	
User	557/2,583 (21.56)	194/1,294 (14.99)	1.56 (1.30-1.86)	<0.001*	1.58 (1.33-1.88)	<0.001*
Alcohol consumption < 1 time a week (n = 4,106)						
Non-user	1,976/2,528 (78.16)	1,297/1,578 (82.19)	1		1	
User	552/2,528 (21.84)	281/1,578 (17.81)	1.29 (1.10-1.51)	0.002*	1.20 (1.03-1.41)	0.023*
Alcohol consumption ≥ 1 time a week (n = 2,689)						
Non-user	1,359/1,729 (78.60)	818/960 (85.21)	1		1	
User	370/1,729 (21.40)	142/960 (14.79)	1.57 (1.27-1.94)	<0.001*	1.75 (1.41-2.17)	<0.001*
SBP < 140 mmHg and DBP < 90 mmHg (n = 1,758)						
Non-user	778/1,034 (75.24)	595/724 (82.18)	1		1	
User	256/1,034 (24.76)	129/724 (17.82)	1.52 (1.20-1.92)	0.001*	1.49 (1.17-1.89)	0.001*
SBP ≥ 140 mmHg or DBP ≥ 90 mmHg (n = 5,037)						
Non-user	2,557/3,223 (79.34)	1,520/1,814 (83.79)	1		1	
User	666/3,223 (20.66)	294/1,814 (16.21)	1.35 (1.16-1.57)	<0.001*	1.33 (1.14-1.54)	<0.001*
Fasting blood glucose < 100 mg/dL (n = 3,700)						
Non-user	1,826/2,322 (78.64)	1,138/1,378 (82.58)	1		1	
User	496/2,322 (21.36)	240/1,378 (17.42)	1.29 (1.09-1.53)	0.004*	1.25 (1.05-1.48)	0.011*
Fasting blood glucose ≥ 100 mg/dL (n = 3,095)						
Non-user	1,509/1,935 (77.98)	977/1,160 (84.22)	1		1	
User	426/1,935 (22.02)	183/1,160 (15.78)	1.51 (1.25-1.82)	<0.001*	1.52 (1.26-1.84)	<0.001*
Total cholesterol < 200mg/dL (n = 4,225)						
Non-user	2,095/2,681 (78.14)	1,285/1,544 (83.23)	1		1	
User	586/2,681 (21.86)	259/1,544 (16.77)	1.39 (1.18-1.63)	<0.001*	1.29 (1.10-1.52)	0.002*
Total cholesterol ≥ 200mg/dL (n = 2,570)						
Non-user	1,240/1,576 (78.68)	830/994 (83.50)	1		1	
User	336/1,576 (21.32)	164/994 (16.50)	1.37 (1.12-1.69)	0.003*	1.49 (1.21-1.84)	<0.001*
CCI scores = 0 (n =21)						

Non-user	8/8 (100.00)	13/13 (100.00)	1		1	
User	0/8 (0.00)	0/13 (0.00)	1.37 (1.21-1.56)	<0.001*	1.36 (1.20-1.55)	<0.001*
CCI score = 1 (n = 5)						
Non-user	1/1 (100.00)	4/4 (100.00)	1		1	
User	0/1 (0.00)	0/4 (0.00)	N/A		N/A	
CCI score ≥ 2 (n = 6,769)						
Non-user	3,326/4,248 (78.30)	2,098/2,521 (83.22)	1		1	
User	922/4,248 (21.70)	423/2,521 (16.78)	N/A		N/A	
Non-GERD user (n = 27,422)						
Non-user	2,915/3,358 (86.81)	1,593/1,750 (91.03)	1		1	
User	443/3,358 (13.19)	157/1,750 (8.97)	1.54 (1.27-1.87)	<0.001*	1.32 (1.10-1.58)	0.003*
GERD user (n = 1,687)						
Non-user	420/899 (46.72)	522/788 (66.24)	1		1	
User	479/899 (53.28)	266/788 (33.76)	2.24 (1.84-2.73)	<0.001*	2.12 (1.73-2.60)	<0.001*
Non-H2 blocker user (n = 4,812)						
Non-user	2,260/2,758 (81.94)	1,783/2,054 (86.81)	1		1	
User	498/2,758 (18.06)	271/2,054 (13.19)	1.39 (1.02-1.91)	0.037*	1.23 (0.91-1.64)	0.173
H2 blocker user (n = 1,983)						
Non-user	1,075/1,499 (71.71)	332/484 (68.60)	1		1	
User	424/1,499 (28.29)	152/484 (31.40)	1.79 (1.41-2.27)	<0.001*	1.67 (1.31-2.14)	<0.001*

Abbreviations: CCI, Charlson Comorbidity Index; SBP, Systolic blood pressure; DBP, Diastolic blood pressure; PPI, proton pump inhibitor; GERD, gastroesophageal reflux disease.

* Significance at $P < 0.05$

† Adjusted for age, sex, income, region of residence, SBP, DBP, fasting blood glucose, total cholesterol, obesity, smoking, alcohol consumption, CCI scores, GERD and H2-receptor antagonist.

Supplementary Table S5. Subgroup analysis of crude and overlap propensity score-weighted odds ratios for the duration of PPI use and mortality in lung cancer participants.

Characteristics	Dead participants (exposure/total, %)	Survived participants (exposure/total, %)	Odd ratios for Lung cancer (95% confidence interval)			
			Crude	P-value	Overlap weighted model †	P-value
Age < 70 years old (n = 3,898)						
< 30 days	2,131/2,313 (92.13)	1,468/1,585 (92.62)	1		1	
≥ 30 days	182/2,313 (7.87)	117/1,585 (7.38)	1.07 (0.84-1.36)	0.577	1.10 (0.86-1.43)	0.445
Age ≥ 70 years old (n = 2,897)						
< 30 days	1,742/1,944 (89.61)	883/953 (92.65)	1		1	
≥ 30 days	202/1,944 (10.39)	70/953 (7.35)	1.46 (1.10-1.94)	0.009*	1.51 (1.15-1.98)	0.003*
Men (n = 5,223)						
< 30 days	3,207/3,521 (91.08)	1,575/1,702 (92.54)	1		1	
≥ 30 days	314/3,521 (8.92)	127/1,702 (7.46)	1.21 (0.98-1.51)	0.077	1.22 (0.99-1.50)	0.060*
Women (n = 1,572)						
< 30 days	666/736 (90.49)	776/836 (92.82)	1		1	
≥ 30 days	70/736 (9.51)	60/836 (7.18)	1.36 (0.95-1.95)	0.095	1.54 (1.03-2.31)	0.038*
Low income (n = 2,947)						
< 30 days	1,796/1,973 (91.03)	894/974 (91.79)	1		1	
≥ 30 days	177/1,973 (8.97)	80/974 (8.21)	1.10 (0.84-1.45)	0.493	1.15 (0.87-1.51)	0.318
High income (n = 3,848)						
< 30 days	2,077/2,284 (90.94)	1,457/1,564 (93.16)	1		1	
≥ 30 days	207/2,284 (9.06)	107/1,564 (6.84)	1.36 (1.06-1.73)	0.014*	1.42 (1.10-1.83)	0.007*
Urban residents (n = 2,845)						
< 30 days	1,603/1,741 (92.07)	1,035/1,104 (93.75)	1		1	
≥ 30 days	138/1,741 (7.93)	69/1,104 (6.25)	1.29 (0.96-1.74)	0.094	1.33 (0.98-1.80)	0.066
Rural residents (n = 3,950)						
< 30 days	2,270/2,516 (90.22)	1,316/1,434 (91.77)	1		1	
≥ 30 days	246/2,516 (9.78)	118/1,434 (8.23)	1.21 (0.96-1.52)	0.106	1.25 (0.99-1.58)	0.062
Underweight (n = 281)						
< 30 days	189/216 (87.50)	62/65 (95.38)	1		1	
≥ 30 days	27/216 (12.50)	3/65 (4.62)	2.95 (0.87-10.07)	0.084	3.08 (1.08-8.74)	0.035*
Normal weight (n = 2,758)						
< 30 days	1,639/1,799 (91.11)	901/959 (93.95)	1		1	
≥ 30 days	160/1,799 (8.89)	58/959 (6.05)	1.52 (1.11-2.07)	0.009*	1.44 (1.06-1.95)	0.019*
Overweight (n = 1,739)						
< 30 days	965/1,048 (92.08)	636/691 (92.04)	1		1	

≥ 30 days	83/1,048 (7.92)	55/691 (7.96)	0.99 (0.70-1.42)	0.976	1.16 (0.79-1.69)	0.450
Obese (n = 2,017)						
< 30 days	1,080/1,194 (90.45)	752/823 (91.37)	1		1	
≥ 30 days	114/1,194 (9.55)	71/823 (8.63)	1.12 (0.82-1.53)	0.482	1.08 (0.78-1.49)	0.652
Non-smoker (n = 2,918)						
< 30 days	1,523/1,674 (90.98)	1,148/1,244 (92.28)	1		1	
≥ 30 days	151/1,674 (9.02)	96/1,244 (7.72)	1.19 (0.91-1.55)	0.211	1.18 (0.89-1.56)	0.258
Past smoker and current smoker (n = 3,877)						
< 30 days	2,350/2,583 (90.98)	1,203/1,294 (92.97)	1		1	
≥ 30 days	233/2,583 (9.02)	91/1,294 (7.03)	1.31 (1.02-1.69)	0.035*	1.39 (1.09-1.79)	0.009*
Alcohol consumption < 1 time a week (n = 4,106)						
< 30 days	2,283/2,528 (90.31)	1,455/1,578 (92.21)	1		1	
≥ 30 days	245/2,528 (9.69)	123/1,578 (7.79)	1.27 (1.01-1.59)	0.039*	1.24 (0.99-1.56)	0.065
Alcohol consumption ≥ 1 time a week (n = 2,689)						
< 30 days	1,590/1,729 (91.96)	896/960 (93.33)	1		1	
≥ 30 days	139/1,729 (8.04)	64/960 (6.67)	1.22 (0.90-1.66)	0.197	1.34 (0.98-1.83)	0.070
SBP < 140 mmHg and DBP < 90 mmHg (n = 1,758)						
< 30 days	928/1,034 (89.75)	668/724 (92.27)	1		1	
≥ 30 days	106/1,034 (10.25)	56/724 (7.73)	1.36 (0.97-1.91)	0.074	1.35 (0.95-1.92)	0.093
SBP ≥ 140 mmHg or DBP ≥ 90 mmHg (n = 5,037)						
< 30 days	2,945/3,223 (91.37)	1,683/1,814 (92.78)	1		1	
≥ 30 days	278/3,223 (8.63)	131/1,814 (7.22)	1.21 (0.98-1.51)	0.080	1.24 (1.00-1.54)	0.055
Fasting blood glucose < 100 mg/dL (n = 3,700)						
< 30 days	2,126/2,322 (91.56)	1,275/1,378 (92.53)	1		1	
≥ 30 days	196/2,322 (8.44)	103/1,378 (7.47)	1.14 (0.89-1.46)	0.297	1.27 (0.98-1.64)	0.069
Fasting blood glucose ≥ 100 mg/dL (n = 3,095)						
< 30 days	1,747/1,935 (90.28)	1,076/1,160 (92.76)	1		1	
≥ 30 days	188/1,935 (9.72)	84/1,160 (7.24)	1.38 (1.05-1.80)	0.019*	1.29 (0.99-1.69)	0.063
Total cholesterol < 200mg/dL (n = 4,225)						
< 30 days	2,428/2,681 (90.56)	1,429/1,544 (92.55)	1		1	
≥ 30 days	253/2,681 (9.44)	115/1,544 (7.45)	1.29 (1.03-1.63)	0.028*	1.19 (0.95-1.50)	0.133
Total cholesterol ≥ 200mg/dL (n = 2,570)						
< 30 days	1,445/1,576 (91.69)	922/994 (92.76)	1		1	
≥ 30 days	131/1,576 (8.31)	72/994 (7.24)	1.16 (0.86-1.57)	0.328	1.40 (1.02-1.91)	0.034*
CCI scores = 0 (n = 21)						

< 30 days	8/8 (100.00)	13/13 (100.00)	1		1	
≥ 30 days	0/8 (0.00)	0/13 (0.00)	1.24 (1.03-1.49)	0.021*	1.27 (1.05-1.52)	0.012*
CCI score = 1 (n = 5)						
< 30 days	1/1 (100.00)	4/4 (100.00)	1		1	
≥ 30 days	0/1 (0.00)	0/4 (0.00)	N/A		N/A	
CCI score ≥ 2 (n = 6,769)						
< 30 days	3,864/4,248 (90.96)	2,334/2,521 (92.58)	1		1	
≥ 30 days	384/4,248 (9.04)	187/2,521 (7.42)	N/A		N/A	
Non-GERD (n = 27,422)						
< 30 days	3,210/3,358 (95.59)	1,694/1,750 (96.80)	1		1	
≥ 30 days	148/3,358 (4.41)	56/1,750 (3.20)	1.39 (1.02-1.91)	0.037*	1.23 (0.91-1.64)	0.173
GERD (n = 1,687)						
< 30 days	663/899 (73.75)	657/788 (83.38)	1		1	
≥ 30 days	236/899 (26.25)	131/788 (16.62)	1.79 (1.41-2.27)	<0.001*	1.67 (1.31-2.14)	<0.001*
H2 blocker (n = 1,983)						
< 30 days	1,329/1,499 (88.66)	410/484 (84.71)	1		1	
≥ 30 days	170/1,499 (11.34)	74/484 (15.29)	0.71 (0.53- 0.95)	0.189	0.80 (0.51 – 1.06)	0.119

Abbreviations: CCI, Charlson Comorbidity Index; SBP, Systolic blood pressure; DBP, Diastolic blood pressure; PPI, proton pump inhibitor; GERD, gastroesophageal reflux disease.

* Significance at $P < 0.05$

† Adjusted for age, sex, income, region of residence, SBP, DBP, fasting blood glucose, total cholesterol, obesity, smoking, alcohol consumption, CCI scores, GERD and H2-receptor antagonist.
